# Effect of Environmental Factors on Low Weight in Non-Premature Births: A Time Series Analysis

**DOI:** 10.1371/journal.pone.0164741

**Published:** 2016-10-27

**Authors:** Julio Díaz, Virginia Arroyo, Cristina Ortiz, Rocío Carmona, Cristina Linares

**Affiliations:** 1 National School of Public Health, Carlos III Institute of Health, Madrid, Spain; 2 Complejo Asistencial Universitario de Salamanca (CAUSA), Salamanca, Spain; Aalto University Helsinki Institute for Information Technology, FINLAND

## Abstract

**Objective:**

Exposure to pollutants during pregnancy has been related to adverse birth outcomes. LBW can give rise to lifelong impairments. Prematurity is the leading cause of LBW, yet few studies have attempted to analyse how environmental factors can influence LBW in infants who are not premature. This study therefore sought to analyse the influence of air pollution, noise levels and temperature on LBW in non-premature births in Madrid during the period 2001–2009.

**Methods:**

Ecological time-series study to assess the impact of PM_2.5_, NO_2_ and O_3_ concentrations, noise levels, and temperatures on LBW among non-premature infants across the period 2001–2009. Our analysis extended to infants having birth weights of 1,500 g to 2,500 g (VLBW) and less than 1,500 g (ELBW). Environmental variables were lagged until 37 weeks with respect to the date of birth, and cross-correlation functions were used to identify explaining lags. Results were quantified using Poisson regression models.

**Results:**

Across the study period 298,705 births were registered in Madrid, 3,290 of which had LBW; of this latter total, 1,492 were non-premature. PM_2.5_ was the only pollutant to show an association with the three variables of LBW in non-premature births. This association occurred at around the third month of gestation for LBW and VLBW (LBW: lag 23 and VLBW: lag 25), and at around the eighth month of gestation for ELBW (lag 6). Leqd was linked to LBW at lag zero. The RR of PM_2.5_ on LBW was 1.01 (1.00 1.03). The RR of Leqd on LBW was 1.09 (0.99 1.19)(*p*<0.1).

**Conclusions:**

The results obtained indicate that PM_2.5_ had influence on LBW. The adoption of measures aimed at reducing the number of vehicles would serve to lower pregnant women's exposure. In the case of noise should be limited the exposure to high levels during the final weeks of pregnancy.

## Introduction

In recent years, various studies have linked exposure during pregnancy to chemical environmental pollutants present in the urban atmosphere to a number of adverse birth outcomes [1–4], including low birth weight (LBW) [5,6]. LBW, defined as any newborn having a weight of less than 2,500g [7], can give rise to lifelong impairments due to respiratory, circulatory and neurological diseases and disorders [8, 9]. The above types of studies tend to have a cohort design [10–12], whereas those that favour a "short-term" methodology based on statistical analysis and, in addition, are generally geographical in nature, are less numerous [13,14]. While cohort studies have the advantage of pinpointing a cause-effect relationship more clearly, they suffer the disadvantage of being more expensive, in that they require a long spatio-temporal follow-up of exposed persons [15]. Recently, time-series studies have been undertaken to address adverse birth outcomes and various environmental factors [16–18]. Time-series studies offer the additional advantage of being able to establish associations, in which individual exposure factors that remain unchanged over time pose no bias [19]. From the standpoint of adverse birth outcomes analysed by both cohort studies and short-term analyses, LBW and premature births, defined as births occurring before the 37th week of pregnancy (PTBs), were considered separately in a great proportion of cases. It is evident that prematurity is related to LBW. Indeed, 60% of births presenting with LBW are PTBs, with prematurity being the leading cause of LBW [20]; even so, few studies have sought to analyse how environmental factors can influence LBW in non-premature births [21,22].

As described in detail previously [18] a growing number of studies have explored the association between air pollutants and LBW, with the most consistent findings showing positive associations with PM_2.5_, PM_10_ and NO_2_ [14,23,24]. Insofar as the underlying pathophysiological mechanism is concerned, PM_2.5_ and ultrafine particles appear to be those which contribute most, linked to a joint action between oxidative stress and pro-inflammatory state. Oxidative stress can be triggered by direct formation of reactive oxygen species coming into contact with free radicals in blood, either because PM_2.5_ is transporting soluble transition metals [25], or because organic materials, such as polycyclic aromatic hydrocarbons (PAHs) (which can also be transported by PM_2.5_), have a high oxidative capacity per se and can enter the cell and affect the mitochondria [26]. Pregnancy physiologically triggers a systemic inflammatory response in the mother. This inflammatory response is enhanced by the above-mentioned processes, leading to the possible occurrence of pre-eclampsia and other processes associated with premature birth [27]. Although many of the studies focus on the effect of PM, there are recent papers which also analyse the effect of NO, ozone and NO_2_ concentrations on adverse birth outcomes and LBW [12,15,28–31].

Furthermore, recent studies indicate that it is not only traditional chemical pollutants present in an urban atmosphere which can influence these adverse birth outcomes, but that other environmental variables, such as traffic noise [13,17,18,32] and temperature in heat and cold waves [16,18,33,34], may also be related to both PTB and LBW. Although the respective biological mechanisms whereby traffic noise and ambient temperature increase the impact on LBW are not clear, there is epidemiological evidence to link them to LBW in the short term [35]. With regard to noise levels, little is known about the association between traffic noise and pregnancy outcomes. It has been hypothesised that stress may affect foetal growth through the endocrine system [36]. Moreover, there is evidence to show increased risk of hypertension among subjects exposed to noise, something that could in turn increase the risk of adverse pregnancy outcomes [1]. An integrated model has recently been published which seeks to explain the short-term effects of traffic noise on various diseases: it includes chronic and acute stress responses which could account for the association between traffic noise and LBW [19].

As described previously [18] in relation to the effect of temperature on adverse birth outcomes, there are few studies that report positive associations [16,18,34]. The results obtained by Kloog et al. point to associations between temperature and birth weight during the last trimester, and between temperature, pre-term delivery and LBW across the entire pregnancy, thus indicating that temperature during pregnancy is associated with lower birth weight and a curtailed gestation period. This study did not differentiate between the effect of heat and cold [37]. Other studies [13] exclusively analyse the effect of heat, and conclude that heat wave temperatures can be a stressful element capable of exerting an influence on low weight. Working along these same lines, Arroyo et al. 2016 [18] analysed the effect of heat and cold, and concluded that only high temperatures are related to prematurity, with this short-term effect being independent of other environmental factors.

Accordingly, the aim of this study was to use a time-series design to analyse the influence of chemical air pollution, noise levels and temperature on LBW among non-premature infants in the city of Madrid, identifying -where possible- the most susceptible periods of exposure during pregnancy by means of significant explanatory lags. The relevance of the results obtained lies in the fact that exposure to these environmental variables can be modified, both at a governmental level by adopting measures targeted at reducing the levels of the environmental risk factors analysed, and at the level of the pregnant women themselves by limiting their personal exposure.

## Methods

### Study population

The city of Madrid is a densely populated metropolitan area situated in the central region of Spain. During the study period, 2001–2009, it had a mean population of 3,164,245 and a birth rate per 1000 population of 10.5, slightly higher than the national rate of 9.1 [38]. The study population consisted of all live singleton births whose mothers resided in the Madrid city area, and whose birth certificates had been filed in the period 1 January 2001 to 31 December 2009. We considered all births that displayed LBW, i.e., newborns weighing less than 2,500g [7], and selected those that corresponded to non-premature births. A non-premature or full-term birth was defined as any birth of more than 37 weeks of gestation [39]. Our analysis also extended to non-premature births with: very low weight (VLBW), defined as any newborn weighing 1,500g to 2,500g; and extremely low weight (ELBW), defined as any newborn weighing less than 1,500 g [40]. Aggregated daily birth counts were collected from the perinatal health databases of public hospitals in Madrid. These daily values were grouped into mean weekly values, with a weekly mean being obtained, which represents the mean weekly value used for analysis purposes.

### Exposure assessment and study design

A time-series analysis was performed to assess the short-term impact of the following environmental variables. Daily mean concentrations (μg/m^3^) of air pollutants, particulate matter less than 2.5 and 10 μm in diameter (PM_2.5_ and PM_10_), tropospheric ozone (O_3_) and nitrogen dioxide (NO_2_), were supplied by the Madrid Municipal Air Quality Monitoring Grid. All measurements were made using the gravimetric method or an equivalent method (beta-attenuation). This network consists of 27 urban background stations across the city. Measurements of acoustic noise pollution were Leqd, equivalent diurnal noise level (7–23 hours) in dB(A), and Leqn, equivalent nocturnal noise level (23–7 hours) in dB(A).

To estimate the daily means of chemical pollutants and noise, each monitor's daily concentration was averaged for that monitor, and then a city-wide average was calculated from all monitors for a given day. All measurements sites included in the study were required to have at least 75% complete information for the study period. Maximum and minimum daily temperatures (°C) in Madrid were furnished by the State Meteorological Agency (AEMET).

These daily values were grouped into mean weekly values for respective variables. Lastly, we controlled for linear trend, seasonality and the autoregressive nature of the series itself.

### Statistical analysis

We decided to fit autoregressive, over-dispersed Poisson regression models of the neonatal outcome variables (controlled for trend and seasonality). Due to their linear relationship, the air pollutants (PM_2.5_, PM_10_, O_3_ and NO_2_) and noise levels (Leqd and Leqn) were introduced into the model as linear components without threshold.However, the relationship between ambient maximum temperature and neonatal outcomes displayed the usual V-shaped pattern already observed in the case of mortality and morbidity variables [41,42]. For analysis purposes, temperature was introduced into the following, two branches: one, in the case of heat, set maximum tempertature at a daily maximum of 34°C; and the other, in the case of cold, set minimum temperature at a daily minimum of -2°C [43].

The variables T_hot_ and T_cold_ were created as follows:

Thot=Tmax−34°C        if Tmax≥34°C

Thot=0        if Tmax<34°C

Tcold=−2°C−Tmin         if Tmin≤ −2°C

Tcold=0         if Tmin>−2°C

Once the daily Thot and Tcal values had been calculated, the weekly averages of these values were computed in order to assess the effect of heat and cold waves on neonatal outcome variables.

To identify the lags at which statistically significant associations between the environmental variables of analysis and the neonatal outcome variables were established, we created variables lagged until 37 weeks with respect to the date of birth, with the aim of ascertaining the effect at a weekly level. In other words, the fact that a variable proved significant at lag 30 would indicate that this significant association occurred 30 weeks before birth, i.e., during week 7 of the pregnancy.

We first established the cross-correlation functions (CFFs) among the residuals of the prewhitened series, with prewhitening being performed using Box-Jenkins methodology [44]. This method consists of modelling each independent environmental variable with an ARIMA model and then applying this model to the dependent variable (LBW), thereby eliminating seasonalities and analogous autocorrelations between the two series. CFFs were established among the residuals of both prewhitened series. The lags at which correlations proved statistically significant were then introduced into the subsequent modelling process (explanatory lags). A model was fitted for each of the variables in relation to the low weight being considered. During the modelling process we only have included the explanatory lags of the environmental variables, excluding those that were not statistically significant lags.

In the case of statistically significant variables (*p*<0.05), the results of the final models were expressed as relative risks (RRs) and their 95% confidence intervals. These RRs were shown for each 10 μg/m^3^increase in the case of the chemical air pollutants, each 1 dB (A) increase in the case of noise levels, and each 1°C increase/decrease in the case of temperature. Based on the respective RR, we then calculated the attributable risk (AR) associated with this increase, by means of the following equation: $\text{AR }=\frac{\text{ RR}-1}{\text{RR}}\text{x}100$ [45].

## Results

During the period 01.01.01 to 31.12.09 there were 298,705 births in Madrid, amounting to a weekly average of 635.5 births across the 470 weeks of the study period. Of this total of weekly births, an average of 7.0 infants per day was born with low weight (7.6%), as can be seen from Table 1. In all, a total of 3,290 births with low weight were registered across the study period, 1,492 of which corresponded to non-premature births: of the latter, 1,176 (83.2%) were VLBW and 236 (16.8%) were ELBW.

**Table 1:** Descriptive statistics of the weekly averages of preterm birth (PB) and low birth weight (LBW) in Madrid in 2001–2009.

Total Births(2001–2009)	Mean	Maximum	Minimum	Standard Deviation
PB	7.5	12.1	3.4	1.5
LBW	7.0	10.1	3.9	1.1

Table 2 shows the descriptive statistics, both for the variables of low weight considered in the analysis for non-premature births, and for the environmental variables used.

**Table 2:** Descriptive statistics of the weekly average variables related to low birth weight in non-premature births and weekly average of the environmental variables in Madrid 2001–2009.

Non premature births	n	Mean	Maximum	Minimum	Standard Deviation
Low birth weight (LBW)	470	3.0	6	1	0.9
Very low birth weight (VLBW)	470	2.5	5	0	0.8
Extremely low birth weight (ELBW)	470	0.5	2	0	0.5
NO_2_ (μg/m^3^))	470	59.3	107.2	30.1	12.5
PM_2.5_ (μg/m^3^)*	314	17.1	41.2	6.6	5.5
PM_10_ (μg/m^3^)	470	32.4	69.4	11.1	10.9
O_3_ (μg/m^3^)	470	35.7	75.0	5.4	16.1
Tmax (°C)	470	20.2	36.7	3.4	8.4
Tmin (°C)	470	10.4	23.7	-2.0	6.3
Leqn (dB(A))	470	59.4	63.0	56.6	0.9
Leqd (dB(A))	470	64.6	67.4	61.6	0.9
Leq24 (dB(A))	470	62.9	65.6	59.9	0.9

* PM_2.5_ concentrations do not start to be measured in Madrid until 2004, so there are fewer observations.

The mean number of weekly births with low weight was 3.0, which meant that 42.9% of births with low weight were not premature and 57.1% were premature.

**Fig 1:**
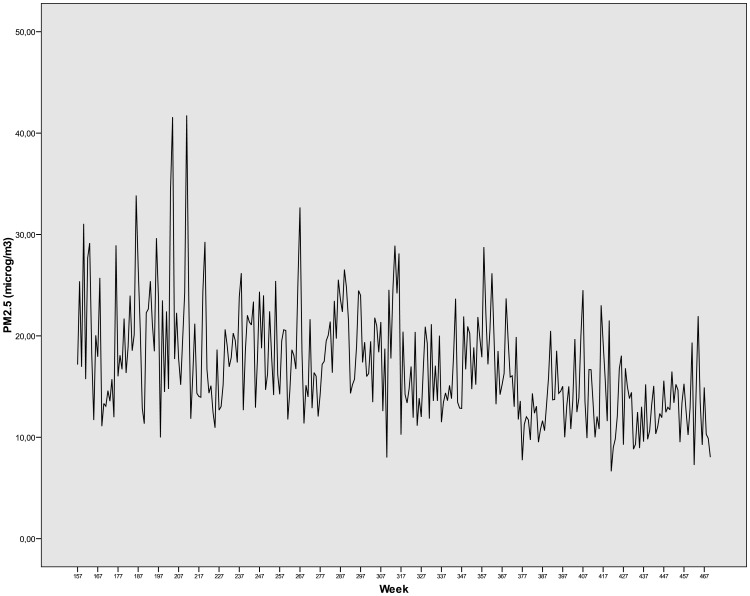
Weekly average of PM_2.5_ concentrations in Madrid 2004–2009.

The WHO guideline values [46] for daily PM_2.5_ concentrations were exceeded on 329 days (10.0%) and daily PM_10_ concentrations were exceeded on 446 days (13.6%) across the study period. In Fig 1, weekly average of PM_2.5_ concentrations in Madrid 2004–2009, can be observed. The WHO guideline values for NO_2_ and ozone (O_3_) were not comparable measurements with the measurements in our study.

**Fig 2:**
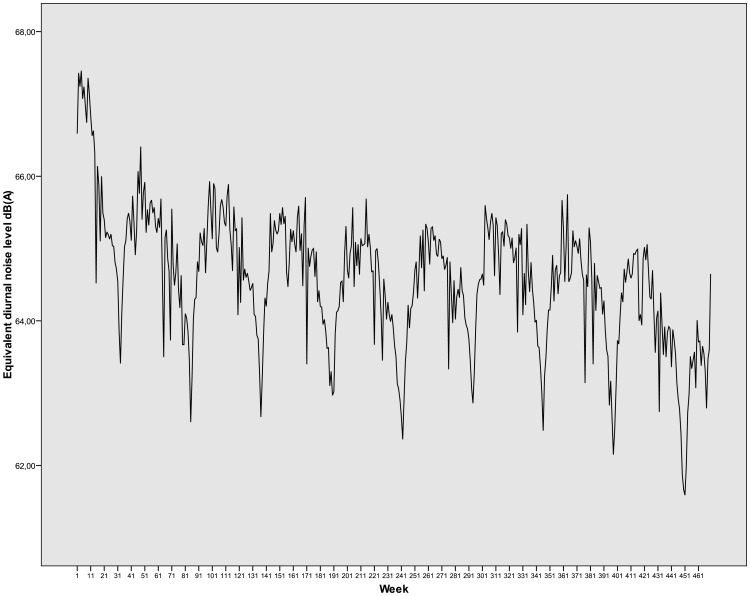
Weekly average of Equivalent Diurnal Noise Levels (Leqd) in Madrid 2001–2009.

Insofar as noise levels were concerned, the mean Leqd value was 64.6 dB(A) with a daily maximum of 69.0 dB(A), and the mean Leqn value was 59.4 dB(A) with a daily maximum of 67.2 dB(A): the WHO guideline values [47] were exceeded on 45% of days and 100% of nights across the study period. Fig 2 shows weekly average of Equivalent Diurnal Noise Levels (Leqd) in Madrid 2001–2009.

In terms of temperature, there were heat waves on 216 days (6.6%) and cold waves on 34 days (1.0%) over the study period. Table 3 shows the explanatory lags at which the pre-determined CFFs showed statistically significant correlations (*p*<0.05) between the different series of independent environmental variables and the variables corresponding to low weight in non-premature births. It should be noted that PM_2.5_ was the sole chemical pollutant to be related to the three selected variables of LBW among non-premature births. These associations occurred: in LBW and VLBW, at around 24 weeks before birth (LBW: lag 23 and VLBW: lag 25), namely, in the third month of pregnancy; and in ELBW, at 6 weeks before birth, namely, around the eighth month of pregnancy. In the case of traffic noise levels, mean diurnal noise level, Leqd, was the only variable to show an association with LBW, and this effect occurred at lag zero, i.e., during the birth week itself.

**Table 3:** Explanatory lags statistically significant (p <0.05) in relation to the low birth weight and the different independent variables of exposure considered in the analysis.

	LBW	VLBW	ELBW
**Explanatory Lags**	PM_2,5_ lag 23Leqd lag 0	PM_2,5_ lag 25	PM_2,5_ lag 6

Table 4 shows the RRs and ARs yielded by Poisson modelling for the above-mentioned explanatory lags. From a quantitative point of view, the effect of PM_2.5_ on the three variables related to low weight displayed very similar ARs, of around 2%. Leqd, however, displayed an appreciably higher AR than that of PM, i.e., around 8%, though this was exclusively for the variable, LBW, and this increase in risk was significant at only *p*<0.1.

**Table 4:** Relative Risks and Attributable Risks obtained from Poisson models.

Low birth weight(LBW)	Very low birth weight (VLBW)	Extremely low birth weight (ELBW)
PM_2,5_ lag 23RR: 1.01 (1.00 1.03)AR = 1.99%Leqd* lag 0RR: 1.09 (0.99 1.19)AR = 8.26%	PM_2,5_ lag 25RR: 1.01 (1.00 1.03)AR = 1.96%	PM_2,5_ lag 6RR: 1.03 (1.00 1.06)AR = 2.91%

*p<0.1

## Discussion

Spain has one of the highest LBW percentages in Europe, after Greece, Portugal and the Czech Republic [48]. With respect to the prematurity rate in Madrid across the study period, 8.2%, this figure is in line with the results obtained for Spain as a whole and shows that Spain has one of the highest prematurity rates in Europe, with values ranging from 5.1% to 10.3% [40]. The results in Table 1, which show that 57.1% of births with low weight correspond to premature births, not only serve to indicate that this percentage is similar to those reported elsewhere [20], but also go to underscore the fact that the leading cause of LBW is prematurity.

Despite the fact that pollution prevention plans in Madrid are activated by exceedance of hourly NO_2_ values set by the European Directive [49], this does not imply compliance with statutory European Union (EU) PM_2.5_ emission values. The reason for this is that EU PM_2.5_ concentrations refer to mean annual values and do thus not come into operation for the purpose of triggering immediate intervention measures, as in the case of NO_2_ [50]. Nonetheless, from the stance of health effects in the Madrid, it is PM_2.5_ concentrations that show the most impact on the population, children and persons over the age of 75 years [51–53]. The fact that PM_2.5_ is the environmental chemical pollutant which displays a statistically significant association with the three indicators of low weight analysed in this study is not in contradiction with other studies in which NO_2_ has been associated with adverse birth variables [6,11,12]. Furthermore, as in the case of NO_2_, there are many papers that link PM_2.5_ to adverse birth variables [10,22,24]. Even so, in multipollutant models, the effect of PM_2.5_ on adverse birth variables is similar to [1] or greater than that of NO_2_ [13]. With respect to our study, it should be stressed that our analysis targeted low weight in both premature and non-premature births, and so the results are not comparable. The variable of chemical pollution linked to prematurity is PM_2.5_ and not NO_2_, something that corroborates our results, since, has already been pointed out, prematurity is the principal cause of low weight. In the few time-series studies on adverse birth outcomes in which PM and NO_2_ were individually introduced, short-term associations were observed with PM, rather than with NO_2_ [16–18].

The association between PM_2.5_ concentrations and both LBW and VLBW is established 23 to 25 weeks before birth, namely, in the third month of pregnancy. This result is in line with those obtained by Arroyo et al. [18] for Madrid. Furthermore, studies undertaken in Connecticut [24] indicate that the strongest association between PM_2.5_ and PTB is established in the first and third months of pregnancy. Its small size and anthropic composition mean that, on being inhaled and reaching the blood circulation, PM_2.5_ particulate matter can cause oxidative stress [25,54,55], a pro-inflammatory [56] and pro-thrombotic state [57] with ensuing placental hypoperfusion. If severe, this will lead to foetal death; and if mild, aside from maternal hypertension [58], this will result in a delay in intrauterine growth due to a reduced supply of oxygen and nutrients [59]. Furthermore, the last month of pregnancy appears to be crucial in terms of the effects that air pollution can have on LBW, giving rise to births with ELBW, as can be seen from the results of our study, in which PM_2.5_ was related to ELBW in the eighth month of pregnancy (lag 6). These results are in line with the study conducted in Beijing [60] which showed that a decrease in PM_2.5_ concentrations in the eighth month of pregnancy translates as higher birth weight. Studies conducted on individual exposures to air pollutants in the different trimesters [61] have shown that the greatest influence on LBW is registered in the third trimester. The same results were obtained in Barcelona by Davdan et al. [13] in relation to PM_2.5_. Maternal factors associated with poorer foetal growth include maternal hypertension and cardiopulmonary disease [60], though it should be mentioned that exposure to air pollution in pregnancy has been associated with an increased risk of hypertensive disorders later in pregnancy [62–64].

Evaluation of the results obtained for the factor of noise exposure shows that noise was the single environmental pollutant that most often exceeded WHO guideline values, both diurnal and nocturnal, across the study period. Recent morbidity-mortality studies in Madrid [65–67] have furnished evidence to show that the impact of noise levels on health is, at the very least, similar to that of PM_2.5_ [67,68], and that such levels are even linked to prematurity, low weight and foetal mortality [17,18]. The finding that the variable "noise" may be related to LBW is in line with the results of other studies which indicate that sound levels have an influence on adverse birth variables [1,13,17,18]. Lastly, the fact that noise would appear to be exclusively related to LBW in the week of birth agrees with the results yielded by previous Madrid-based studies [17,18] which show noise to be related to both LBW and the number of premature births at the date of birth itself. In other words, acting through acute stress mechanisms [19], noise can induce birth after 37 weeks of gestation, and this can in turn lead to a newborn having LBW (though not ELBW).

With respect to the variable "temperature", the number of heat and cold waves which occurred in Madrid across the study period (216 and 34 respectively) and their effects on population health [43], including prematurity [18], justify their inclusion in the analysis as two variables with independent effect [37], since, even though the number of cold waves was very much lower than that of heat waves, in Spain daily mortality attributable to cold waves is higher than that attributable to heat [69]. The reason why temperature in heat and cold waves is not shown to be related to LBW resides in the fact that an underlying stress mechanism is once again at work [70], with the result that its effect is fundamentally seen in the final stages of pregnancy, causing prematurity in the birth [35]. By eliminating premature births from our LBW study, this short-term effect was likewise eliminated and does thus not appear as being related to LBW in non-premature births.

The nature of our longitudinal study renders it impossible to quantify to what precise degree low weight is associated with an increase in PM_2.5_ concentrations or dB(A), as has been done by other studies [4,60]. What is does allow for, however, is to establish the increase in risk, expressed as RR, of being born with low weight, between an infant who is exposed to an increase of 10μg/m^3^/m^3^ in PM_2.5_ or of 1 dB(A) in Leqd and another who is not so exposed. The 2% increase in risk of LBW for every 10μg/m^3^increase in PM_2.5_ was of the same order as that reported by similar studies [22,24]. Once again, noise, as measured by the indicator Leqd, was shown to have a greater impact on LBW than did PM_2.5_, as was also the case in premature births in Madrid [18] and even foetal mortality [17].

With respect to the limitations of this study, an ecological study such as ours suffers from the ecological fallacy. Furthermore, an acknowledged limitation of all studies of environmental data as it is previously described in [71] is that measurements from stationary outdoor monitors may not represent individual exposures, though relatively crude, ambient measures are often the most feasible measure of exposure in terms of reducing both research costs and discomfort for the study participant [72]. Not only do air-pollutant concentrations and noise levels have different spatial distributions, but the degree to which outdoor levels reflect indoor levels also varies. This leads to different degrees of measurement error and, thus, of power for each of these, and may influence which associations are detected. The advantage of this type of time series design versus for example cohort design, is that they can be used to detect what variables are associated and identify periods of exposure more sensitive for pregnant. These studies are less expensive and reinforce the results obtained in cohort designs. Though the methodology used enable us to found the week in which there is a statically significant association, from a biologically point of view it is coherent to interpret these results in a wider sense. The objective is can establish the sensitive windows of pregnancy in which the fetus is more susceptible to each pollutant among the analyzed.

The results obtained in this study indicate that during the study period, chemical pollution (PM_2.5_) had an important influence on LBW in Madrid. In such an urban atmosphere, the source of PM_2.5_ is linked to the number of diesel vehicles. Hence, the adoption of measures at a governmental level to reduce the number of these vehicles would result in a lower exposure of pregnant women to this pollutant and, by extension, in a possible decrease in the number of infants with LBW. In the case of noise, 80% of which is traffic-generated in a city such as Madrid [43], the above governmental measures should be supplemented by adopting individual purpose-designed measures aimed at limiting exposure to high noise levels during the final week’s pregnancy.

## Supporting Information

S1 File Dataset used for the analysis. (SAV)
